# Internal traction in endoscopic full-thickness resection for gastric subepithelial lesions arising from the muscularis propria: Comparative study

**DOI:** 10.1055/a-2544-2572

**Published:** 2025-06-12

**Authors:** Jun Li, Xiaojia Hou, Kan Chen, Kangsheng Peng, Chao Huang, Feng Liu

**Affiliations:** 1 278245 Digestive Endoscopy Center, Shanghai Tenth People's Hospital, Shanghai, China; 2 278245 Department of Gastroenterology, Shanghai Tenth People's Hospital, Shanghai, China

**Keywords:** Endoscopy Upper GI Tract, Precancerous conditions & cancerous lesions (displasia and cancer) stomach, Endoscopic resection (ESD, EMRc, ...), Endoscopic ultrasonography, Subepithelial lesions

## Abstract

**Background and study aims:**

Effective tissue traction is crucial for gastric endoscopic full-thickness resection (EFTR) to ensure a clear visual field for the dissection site. We aimed to evaluate the effectiveness of internal traction using a novel clip-with-spring device in assisting gastric EFTR.

**Patients and methods:**

Twenty-six patients with gastric subepithelial lesions from the muscularis propria were enrolled for internal traction-assisted EFTR (IT-EFTR) and 26 patients for non-assisted EFTR (NA-EFTR) were enrolled as controls.

**Results:**

Average tumor size was 1.5 ± 0.4 cm. All EFTRs were completed successfully with an average total procedure time of 62.4 ± 43.0 minutes and perforation time of 37.2 ± 29.9 minutes. En bloc resection was achieved in 50 patients (96.2%). IT-EFTR significantly improved serosa exposure score (3.4 ± 0.9 vs. 1.9 ± 0.7, *P* <0.001) and shortened total procedure time (33.0 ± 21.8 vs. 91.8 ± 38.6 min, *P* <0.001) and perforation time (19.0 ± 18.8 vs. 55.5 ± 27.8 min, *P* <0.001) compared with NA-EFTR. There were no significant differences in complication rates between the two groups. However, visual analogue score after the procedure was significantly lower (4.2 ± 1.0 vs. 4.7 ± 0.7, *P* =0.037) and postoperative hospital stay (3.7 ± 2.1 vs. 4.8 ± 1.3, *P* =0.038) was significantly shorter in the IT-ERTR group than in the NA-EFTR group.

**Conclusions:**

Internal traction using the novel clip-with-spring device could significantly improve safety and efficacy of gastric EFTR in the distal stomach.

## Introduction

Endoscopic full-thickness resection (EFTR) is an advanced endoscopic technique that after more than two decades of development, is now widely applied in resection of gastric subepithelial lesions (SELs) originating from the muscularis propria (MP) 1 . Performing EFTR is challenging and requires a high level of endoscopic skills and experience. Effective tissue traction to ensure a clear visual field for the dissection site is crucial during EFTR. In the past few years, many studies have reported on use of external traction by clip-with-thread or clip-with-snare device in assisting EFTR 2 3 4 . These techniques were effective for resection of lesions, especially in the gastric fundus 5 6 7 . However, traction effects were less satisfactory for lesions in the distal stomach. In addition, external traction techniques require endoscope withdrawal and are relatively complex to apply. In the present study, we introduced an internal traction strategy by using a novel clip-with-spring device to assist EFTR for resection of MP-SELs in the distal stomach. We aimed to evaluate effectiveness and safety of this technique and provide vital evidence to guide future clinical practice.

## Patients and methods

### Study design and patient enrollment

This was a single-center, retrospective, cohort study. A total of 26 consecutive patients with MP-SELs in the distal stomach were enrolled for internal traction-assisted EFTR (IT-EFTR group) from January to September 2023 in Shanghai Tenth People’s Hospital. Another 26 patients with MP-SELs in the distal stomach who had undergone non-assisted EFTR (NA-EFTR group) from January to December 2022 in our hospital were involved as controls. For all patients, inclusion criteria were: age 35 to 80 years old; proven diagnosis of MP-SELs by common gastroscopy, endoscopic ultrasound, and contrast-enhanced computed tomography (CT); location in the lower two-thirds of the corpus or the antrum; and no regional lymph node or distant metastasis demonstrated on CT or MRI. Exclusion criteria were: lesions with absolutely extraluminal growth pattern or high-risk features of malignancy that were not amenable to endoscopic treatment; location at the fundus or in the upper third of the corpus that could be applied the clip-with-thread technique; anticoagulant/antiplatelet agents that could not be suspended, and severe comorbidities or poor conditions such that the patient could not tolerate the operation. The study was approved by the Ethics Committee of Shanghai Tenth People’s Hospital and written informed consent was obtained from all patients.

### EFTR procedure

Internal traction-assisted EFTR using the clip-with-spring device.Video 1

**Fig. 1:**
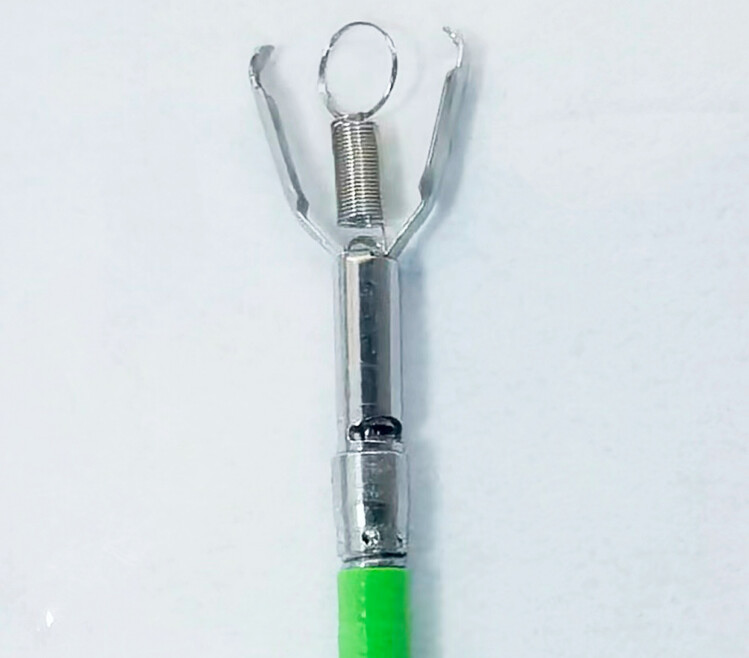
The novel clip-with-spring device consists of a metal clip and a 5-mm spring fixed between the two claws of the clip.

**Fig. 2:**
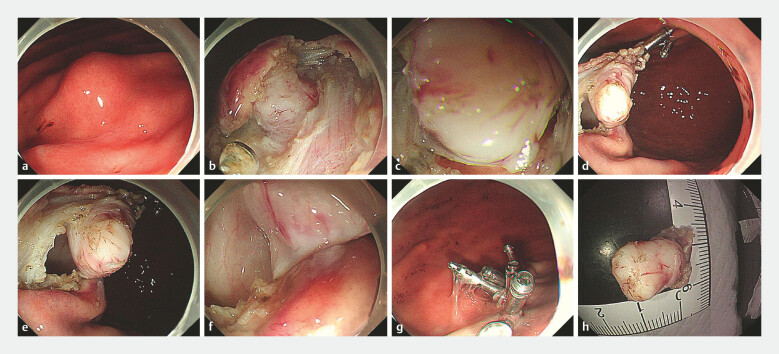
Procedures of internal traction-assisted EFTR using the clip-with-spring device. a Endoscopic view of the gastric SEL. b Mucosa incision and submucosal dissection to expose the tumor body. c Full-thickness resection with poor visualization of the dissection site. d Internal traction using the clip-with-spring device was applied. e,f Resection became easy under countertraction. g After resection of the lesion, the mucosal defect was closed with metal clips; h. The gross specimens.

All patients underwent EFTR under general anesthesia with intubation in left lateral position under carbon dioxide insufflation. For IT-EFTR, the procedures were performed as follows. First, the lesion was identified and submucosal injection was performed using sterile normal saline premixed with 1% indigo carmine. Next, an incision was made in the mucosa on the oral side of the lesion margin and initial submucosal dissection was performed to reveal the tumor. Submucosal excavation as deep as the seromuscular layer around the tumor body then was performed. In cases in which it was difficult to expose the dissection site between the tumor and the seromuscular layer, attempts by NA-EFTR were allowed for no longer than 10 minutes, or the IT-EFTR would be conducted. The clip-with-spring device has been described in our previous studies 8 9 . It consists of a metal clip and a 5-mm long spring with one end fixed between the two claws and the other end shaped like a ring ( Fig. 1 ). When in use, it was inserted through the biopsy channel and anchored on the proximal edge of the resected mucosa above the lesion. Another metal clip was used to grasp the ring of the spring, pull it to the opposite gastric wall, and anchor it on the mucosa. Traction direction could be adjusted by changing the anchoring site on the opposite gastric mucosa 9 . After traction was applied, the seromuscular layer under the tumor could be clearly visualized. Full-thickness resection of the tumor and the surrounding MP layer and serosa then was performed. Gastric wall defects were closed using metal clips. After resection, the specimen together with the clip-with-spring device was retrieved using grasping forceps. The IT-EFTR procedure is shown in Fig. 2 and Video 1 .

### Post-EFTR management

All patients were kept fasted for at least 48 hours after the procedure and given intravenous (IV) fluids, proton pump inhibitors (PPIs), and prophylactic antibiotics. If no signs of bleeding or perforation occurred after 48 hours, clear fluids and subsequent soft diets were introduced gradually. Abdominal pain was evaluated immediately after the procedure and daily thereafter using the visual analogue score (VAS), which ranged from 0 (no pain) to 10 (severe pain). If the VAS score was above 5 and symptoms such as abdominal distension or signs of peritonitis developed, a thoracoabdominal CT was performed to rule out perforation. Endoscopic examination was repeated in cases of hematemesis or melena and endoscopic hemostasis was carried out if necessary. Oral PPIs were prescribed routinely for 2 months after discharge. Criteria for discharge were as follows: completely normal for infection indicators including normal blood test, C-reactive protein and procalcitonin; stable hemoglobin; normal temperature (< 37.2°C; no obvious symptoms such as stomachache (VAS score ≤ 2), hematemesis or melena emerged after soft diets; and no significant signs of any other serious discomfort related to EFTR.

### Definitions

**Fig. 3:**
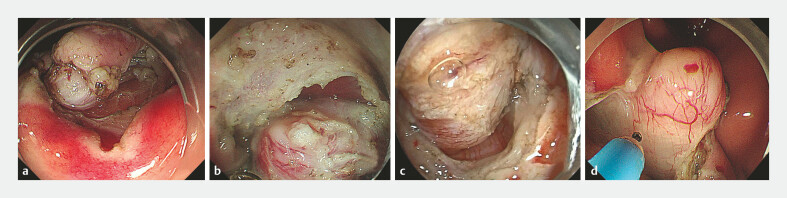
Different serosa exposure score during EFTR. a 1, no serosa could be seen. b 2, <1/2 serosa of the tumor body could be seen. c 3, More than half but not the entire serosa of the tumor body could be seen. d The entire serosa area of the tumor body with adjacent serosa could be seen.

Procedure time was defined as time between submucosal injection of the first dot and last withdrawal of the endoscope. Perforation time was defined as time between initial creation of the iatrogenic perforation and complete closure of the defect. En bloc resection was defined as intact excision of the tumor in one piece without fragmentation. Intraoperative bleeding was defined as oozing or pulsating bleeding that necessitated use of hemostatic forceps during the procedure. Delayed bleeding was defined as hematemesis or melena with decrease in hemoglobin level > 2 g/dL after EFTR. Serosa exposure score was classified as 1, 2, 3, and 4 depending on whether no serosa, less than half the serosa of the tumor body, more than half but not the entire serosa of the tumor body, or the entire serosa area of the tumor body with adjacent serosa, respectively, could be seen during EFTR ( Fig. 3 ).

### Statistical analysis

Statistical analyses were performed using SPSS 23.0 software (SPSS Inc., Chicago, Illinois, United States). Continuous variables were presented as mean ± standard deviation and were compared using unpaired Student’s *t* test. Comparison of categorical variables was performed using *Χ ^2^* tests or Fisher’s exact test. A two-sided *P* ≤ 0.05 was considered statistically significant.

## Results

### Main features of study population

The study involved 18 males (34.6%) and 34 females (65.4%) with a mean age of 59.6 ± 8.9 years (range 38–78 years). Average tumor size was 1.5 ± 0.4 cm (range 0.9–2.5 cm). Forty-eight tumors (92.3%) were located in the corpus and four (7.7%) in the antrum. Twenty tumors (38.5%) presented with a partly extraluminal growth pattern, whereas 32 tumors (61.5%) showed a predominantly intraluminal growth pattern. All EFTR procedures were completed successfully with an average total procedure time of 62.4 ± 43.0 minutes and perforation time of 37.2 ± 29.9 minutes. All specimens were retrieved orally and histopathological evaluation revealed that 40 tumors were gastrointestinal stromal tumors (GISTs) (76.9%) and 12 tumors were leiomyomas (23.1%). All GISTs were low or very low risk according to National Comprehensive Cancer Network Guidelines 10 . En bloc resection was achieved in 50 patients (96.2%).

### Comparisons of clinical characteristics and therapeutic outcomes

Comparisons of clinical characteristics and therapeutic outcomes are listed in Table 1 . Patients in the two groups had no significant differences in sex, age, tumor diameter, tumor location, tumor growth pattern, en bloc resection rate, or histopathology.

**Table:** **Table 1** Comparisons of clinical characteristics and therapeutic outcomes between patients in the two groups.

Variables	IT-EFTR group (n = 26)	NA-EFTR group (n = 26)	*P* value
Sex (M/F)	10/16	8/18	0.560
Age, mean ± SD, years	60.9 ± 10.0	58.4 ± 7.7	0.316
Maximum tumor diameter, mean ± SD, cm	1.5 ± 0.4	1.6 ± 0.4	0.207
Tumor location (corpus/antrum)	24/2	24/2	1.000
Intraluminal growth pattern (predominantly/partly)	15/11	17/9	0.569
En bloc resection, n *(* %)	26 (100)	24 (92.3%)	0.490
Procedure time, mean ± SD, min	33.0 ± 21.8	91.8 ± 38.6	**<0.001**
Perforation time, mean ± SD, min	19.0 ± 18.8	55.5 ± 27.8	**<0.001**
Serosa exposure score			
mean ± SD	3.4 ± 0.9	1.9 ± 0.7	**<0.001**
< 3/≥ 3	6/20	22/4	**<0.001**
VAS score after operation, mean ± SD	4.2 ± 1.0	4.7 ± 0.7	**0.037**
Histopathological type (leiomyoma/GIST)	7/19	5/21	0.510
Postoperative hospital stay, mean ± SD, d	3.7 ± 2.1	4.8 ± 1.3	**0.038**
Complications, n *(* %)			
Intraoperative bleeding, n *(* %)	5 (19.2)	5 (19.2)	1.000
Delayed bleeding, n *(* %)	1 (3.8)	2 (7.7)	1.000
Fever (> 38°C, n *(* %)	2 (7.7)	3 (11.5)	0.638
EFTR, endoscopic full-thickness resection; GIST, gastrointestinal stromal tumor; IT, internal traction; NA, non-assisted; SD, standard deviation.			

IT-EFTR significantly improved the serosa exposure score during the procedure when compared with NA-EFTR (3.4 ± 0.9 vs. 1.9 ± 0.7, *P* < 0.001). The percentage of patients with a serosa exposure score of 3 or 4 in the IT-EFTR group was significantly higher than that in the NA-EFTR group (76.9% vs 15.4%, *P* < 0.001). Total procedure time was significantly shorter in the IT-EFTR group than in the NA-EFTR group (33.0 ± 21.8 vs. 91.8 ± 38.6 min, *P* < 0.001). IT-EFTR also significantly shortened perforation time when compared with NA-EFTR (19.0 ± 18.8 vs. 55.5 ± 27.8 min, *P* < 0.001) ( Table 1 ).

### Complications

Ten patients (19.2%) suffered intraoperative bleeding and three patients (5.8%) suffered delayed bleeding. These cases were all successfully managed with endoscopic hemostasis. Five patients (9.6%) had transient fever above 38°C after EFTR, which was managed with upgraded IV antibiotics. Rates of these complications were not significantly different between the two groups ( Table 1 ). However, VAS scores after the procedure were significantly lower in patients in the IT-ERTR group than in the NA-EFTR group (4.2 ± 1.0 vs. 4.7 ± 0.7, *P* = 0.037). None of the patients presented with delayed perforation, massive bleeding, or any other serious complications. All patients were discharged with no severe complications after a mean postoperative hospital stay of 4.3 ± 1.8 days. Postoperative hospital stays were significantly shorter in the IT-EFTR group than in the NA-EFTR group (3.7 ± 2.1 vs. 4.8 ± 1.3, *P* = 0.038).

### Follow-up

All patients underwent surveillance endoscopy within 6 months of the procedure. Wound healing was satisfactory in all cases with no residual or recurrent tumors observed.

## Discussion

EFTR is a more challenging endoscopic technique than other endoscopic techniques such as endoscopic submucosal dissection. After full-thickness incision, it is difficult to maintain intraluminal gas in the digestive tract, resulting in poor exposure of the dissection field and inability to clearly observe blood vessels, particularly on the serosa side. In addition, the lesion is more likely to prolapse into the abdominal side due to the effect of gravity on the detached lesion and the pressure difference between the inside and outside of the digestive tract, which makes the procedure difficult to continue. Various auxiliary traction techniques are expected to reduce the difficulty and risk of EFTR and increase effectiveness. Traction may help to pull the lesion into the gastric cavity, expose the lesion, and make the dissection site clear, which could facilitate the process. In addition, traction may help to quickly identify bleeding points during EFTR to allow prompt hemostasis and this method may prevent accidental injury of extraluminal vessels. Traction also may help prevent specimens from dropping into the abdominal cavity. Although various traction methods using adjunctive devices have been developed in the past few years, comparative studies concerning usefulness and efficacy of traction devices in assisting EFTR remain far from sufficient.

In the present study, for the first time, we reported an internal traction strategy using a novel clip-with-spring device in assisting EFTR for gastric MP-SELs. The results showed that this technique significantly improved dissection vision, represented by the serosa exposure sore, and consequently significantly shortened total procedure and perforation times. Although complication rates were not significantly different between the two groups, this technique significantly reduced patient discomfort after the procedure. This may be explained by the shorter perforation time, which meant less leakage of gas and gastric fluids into abdominal cavity. Less time for postoperative in-hospital observation also contributed to significantly shorter postoperative hospital stays in the traction group than in the non-traction group. Our study demonstrated great usefulness and efficacy of this technique in assisting EFTR.

Compared with other previously reported techniques, this technique has many advantages. First, it is sterile and ready to use without special preparation and endoscope withdrawal, making it safer and more convenient when compared with thread, rubber-band, or snare-based traction methods 2 3 4 5 6 7 . Second, traction force could be applied in any direction by using the clip-with-spring device. In contrast, the direction of the clip-with-thread technique is limited to the direction in which the line is pulled 5 6 7 . Third, the traction direction of our clip-with-spring technique could be easily adjusted during the procedure, if necessary, without changing patient position 9 . If towing is in the wrong direction, the ring of the clip-with-spring device could be removed from the first anchoring site using a forceps and re-anchored to a second site using another clip. Traction force could be controlled by suction or inflation with intraluminal gas. In comparison, the gravity or magnetic-based traction strategy usually involves position change and/or relatively fixed traction force 11 . Finally, this technique is more cost-effective when compared with some expensive dedicated device such as OTSC 12 , FTRD 13 14 , or robotics 15 16 .

Concerning the clip-with-spring device, Sakamoto et al previously reported on a similar device named the S–O clip, which is a clip with a 5-mm spring plus a 4-mm nylon loop on one of the clip claws 17 . This device is convenient to use and could provide internal traction in any direction. However, it was only described for assisting ESD of gastrointestinal superficial lesions 18 19 20 . No comparison study has been published to prove the efficacy of this device in EFTR for gastric SELs. Whether the internal traction strategy using such a clip-with-spring device may improve safety and efficacy of gastric EFTR remained unknown before our study. In addition, our novel clip-with-spring device has some modifications. The spring in our device is shorter when compared with the S-O clip, enabling continuous tension throughout the EFTR process. The metal ring at the top of the spring is more convenient to grasp with another clip compared with the nylon loop of the S-O clip, which is prone to deformation due to lack of rigidity.

Our study had several limitations. First, all EFTR procedures were performed by a single highly experienced endoscopist (Feng Liu) in a single center, which may impact generalizability of the results. Because timing of EFTR differed between the IT-EFTR and NA-EFTR groups, that may have impacted the technical maturity of the endoscopist. Second, we included a relatively small sample of participants. The steps in gastric EFTR can be affected by various factors. Specialized lesion locations, differences in gastric cavity configuration, and vascular malformations inevitably are associated with variation in EFTR procedure times and risk of complications in patients undergoing the procedure. Third, we did not include patients with MP-SELs in the proximal stomach due to well-established application of the clip-with-thread technique in treating such lesions. Comparisons between the clip-with-thread and clip-with-spring method could not be conducted. Finally, it was not possible to perform double-blinding. However, given the diverse lesions and equivalent baseline characteristics in the IT-EFTR and NA-EFTR groups, our results provide preliminary evidence of the significant potential for IT-EFTR using the novel clip-with-spring device. Large-scale randomized, controlled studies are warranted for further investigation.

### Conclusions

In summary, internal traction using the novel clip-with-spring device could significantly improve safety and efficacy of EFTR for MP-SELs in the distal stomach.
